# Early Initiation of novel hormonal therapy is associated with improved survival in synchronous bone-metastatic hormone-sensitive prostate cancer: a retrospective cohort study from China

**DOI:** 10.3389/fonc.2026.1719338

**Published:** 2026-04-01

**Authors:** Yize Guo, Yuefeng Jia, Yunduo Fan, Wei Zhang, Yanjiang Li, Yao Li

**Affiliations:** 1 Department of Urology, The Affiliated Hospital of Qingdao University, Qingdao, Shandong, China; 2 Department of Cardiology, The Affiliated Hospital of Qingdao University, Qingdao, Shandong, China

**Keywords:** high disease volume, novel hormonal agents, prostate cancer, risk stratification, synchronous bone metastasis

## Abstract

**Background:**

The incidence and prognosis of synchronous bone-metastatic hormone-sensitive prostate cancer (SBM-HSPC) in contemporary China remain unclear. This study aimed to determine its current prevalence, characterize patient profiles, and identify independent prognostic factors.

**Methods:**

We conducted a retrospective cohort study of patients with SBM-HSPC diagnosed at a major tertiary center in China (2017-2023). Demographic, clinicopathological, treatment, and outcome data were analyzed. Overall survival (OS) was evaluated using Kaplan-Meier and Cox regression. A landmark analysis (3- and 6-month) was employed to assess the association between early treatment initiation and OS, mitigating immortal time bias.

**Results:**

The incidence of SBM-HSPC was 6.09%. The cohort presented with high-risk features: 93.1% had Gleason score ≥8,and 83.7% had high-volume disease (HVD). The median OS was 43 months. Multivariate analysis identified HVD as an independent risk factor (HR = 2.37, P = 0.012) and age 60–74 years as protective (HR = 0.52, P = 0.040) compared to age <60. Landmark analysis demonstrated that initiation of novel hormonal agents (NHA) within 3 months was associated with a 45% reduction in mortality risk (HR = 0.55, P<0.001), an association sustained at 6 months. In contrast, early use of docetaxel or bone-targeting agents was not associated with improved OS.

**Conclusion:**

The incidence of SBM-HSPC in this Chinese cohort aligns with data from developed healthcare systems. HVD is a key prognostic determinant. Early initiation of NHA therapy is strongly associated with a significant survival benefit, underscoring the critical need for timely, intensive systemic treatment in this high-risk patient population.

## 1 Introduction

Prostate cancer (PC) ranks as the second most prevalent malignancy among men globally, with approximately 1.5 million new cases annually (1). Prognosis exhibits marked heterogeneity: patients with localized early-stage disease achieve 10-year survival rates exceeding 99%, whereas metastatic progression portends universally poor outcomes and is considered incurable despite multimodal therapeutic strategies (2, 3). However, the treatment paradigm is evolving with the recognition of oligometastatic prostate cancer. Emerging evidence suggests that cytoreductive radical prostatectomy (cRP) may improve survival in selected patients by reducing tumor burden and interrupting metastatic reseeding (4).Metastatic prostate cancer (mPC) is classified into synchronous or metachronous subtypes based on metastasis detection timing relative to initial diagnosis (5). Synchronous mPC accounts for approximately 10% of cases worldwide and is characterized by heightened aggressiveness, accelerated progression to castration resistance, and inferior survival outcomes (5–8).

Bone represents the most frequent metastatic site, frequently triggering skeletal-related events (SREs) such as pathological fractures, spinal cord compression, and requirements for palliative radiotherapy or bone surgery (9). STAMPEDE, LATITUDE and PEACE-1 studies have elevated abiraterone acetate and prednisone to the first-line treatment position for bone metastatic prostate cancer patients (10–12). Patients diagnosed with bone metastasis at initial presentation are treatment-naïve and hormone-sensitive; thus, we define this cohort as synchronous bone-metastatic hormone-sensitive prostate cancer (SBM-HSPC). Historically, multicenter studies from China reported alarmingly high SBM-HSPC rates of up to 54%, vastly exceeding global averages (13). This discrepancy may relate to the evolution of prostate-specific antigen (PSA) screening practices: following the U.S. Preventive Services Task Force (USPSTF) recommendations against routine screening in 2008 and 2012 due to overdiagnosis concerns, mPC incidence subsequently rebounded in the United States (14). Despite ongoing debate regarding PSA screening’s clinical utility, the Chinese Urological Association(CUA)has endorsed its implementation since 2011, given China’s high metastatic burden. Nevertheless, the impact of this policy shift on SBM-HSPC incidence remains unclear. To address this knowledge gap, we conducted a comprehensive analysis of data from patients diagnosed with SBM-HSPC at The Affiliated Hospital of Qingdao University between 2017 and 2023, with the objectives of determining its contemporary prevalence, characterizing clinicopathological features, and identifying independent prognostic factors.

## 2 Methods

### 2.1 Study design and ethical approval

This single-center, retrospective cohort study consecutively enrolled patients with SBM-HSPC at The Affiliated Hospital of Qingdao University (Three-tier referral center, with four campuses distributed across regions with significant GDP disparities) between January 2017 and December 2023. The study protocol was approved by the Institutional Ethics Review Board of the hospital (No. QYFY WZLL 30427). Informed consent was waived due to the retrospective nature of the study. All procedures were conducted in accordance with the principles of the Declaration of Helsinki.

### 2.2 Patient selection criteria

Inclusion criteria were: (1) initial diagnosis of prostate cancer confirmed by histopathology; (2) radiologically confirmed bone metastases (based on ECT and/or CT and/or MRI); (3) treatment-naive and hormone-sensitive at the time of initial diagnosis. Exclusion criteria included: (1) non-SBM-HSPC (metachronous metastases under treatment); (2) concurrent other malignant tumors. The final cohort comprised 203 patients (28 excluded due to loss to follow-up).

### 2.3 Variable definitions

Demographic characteristics: Age at diagnosis, body mass index (BMI), smoking history(smoke), alcohol consumption(drink), and comorbidities [hypertension, diabetes, chronic heart disease (CHD)].

Tumor characteristics: Histological type [based on WHO 2022 criteria: prostatic acinar adenocarcinoma (PAA), prostatic ductal adenocarcinoma (PDA), neuroendocrine prostate cancer (NEPC)], Gleason score (≤6, 7, 8–10), baseline prostate-specific antigen (PSA) level (≤4, 4–10, 10–20, 20–100, >100 ng/mL), primary tumor stage (AJCC 8th edition TNM staging), visceral metastasis (yes/no), axial bone involvement.

Assessment of Bone Metastases:(1) Imaging and Diagnosis: Baseline bone metastases were assessed using a combination of anatomical and functional imaging. All patients underwent a 99mTc-MDP whole-body bone scintigraphy (ECT) to evaluate the entire skeleton. Suspicious lesions on bone scan were confirmed as metastases by correlating with corresponding structural abnormalities (osteolytic, osteoblastic, or mixed) on contemporaneous diagnostic CT or MRI scans obtained as part of standard staging. A lesion was considered a confirmed bone metastasis only if both modalities (increased uptake on ECT + structural change on CT/MRI) were concordant; (2) Quantification and Classification: The total number of bone metastases was counted based on the confirmed lesions on the bone scan. Disease extent was categorized as axial (vertebral bodies and pelvis) or beyond-axial (all other bones) according to the CHAARTED criteria; (3) Image Review: All imaging studies were reviewed independently by two radiologists specializing in genitourinary or nuclear medicine imaging. Discrepancies were resolved by consensus or by a third senior reviewer; (4) Timing: All imaging for baseline metastasis assessment was completed within 4 weeks before the start of the following treatment.

Disease volume (DV): Defined per CHAARTED criteria using conventional imaging. High-volume disease was defined as either: (1) ≥4 bone lesions with at least one beyond the axial skeleton (spine/pelvis), or (2) presence of visceral metastases. Metastatic status not meeting high-volume criteria was classified as low-volume (15).

Definition and Ascertainment of SREs: (1) SREs Definition - Skeletal-related events were defined as the first occurrence of any of the following after bone metastasis diagnosis: pathologic fracture, spinal cord compression, radiation therapy to bone, or surgery to bone. Bone pain or analgesic use alone did not qualify as an SRE; (2) Data Capture: Potential SREs were identified through a structured retrospective review of all electronic medical records, including radiology, radiation oncology, surgical, and clinical oncology notes; (3) Event Adjudication: All potential events were independently reviewed and validated by two study clinicians using the pre-specified criteria. Disagreements were resolved by consensus or, if needed, by a third senior investigator. The date of the first confirmed SRE was recorded for analysis.

Treatment modalities: Androgen Deprivation Therapy (ADT); docetaxel chemotherapy; Novel Hormone Agent (NHA:abiraterone/enzalutamide/apalutamide/darolutamide); bone-protecting agents [bisphosphonates (BPs)/Anti-RANKL monoclonal antibody (RANKL)]; analgesic regimens (no analgesic required, NSAIDs monotherapy, weak opioids, single-agent strong opioids, combination strong opioid therapy).

The follow-up duration was calculated from the initial diagnosis date to the date of either the primary endpoint event (e.g., death) or the last confirmed follow-up (censoring). Patients who were event-free at the cutoff date or lost to follow-up were censored at their last known contact date.

### 2.4 Statistical analysis

The analysis of this study adopts complete case analysis. When constructing the final multivariate Cox regression model and milestone analysis cohort, cases with missing values of any analysis variables are excluded from this specific analysis. The completeness of baseline characteristics of this cohort was high, and the missing rate of main variables was less than 5%. Continuous variables were reported as median (IQR) or mean ± SD based on normality testing; categorical variables as frequencies (percentages). Group comparisons utilized t-tests or χ² tests. Survival curves were plotted via Kaplan-Meier method and compared with log-rank tests. Significant variables from univariate analysis (p < 0.2) were included in multivariate Cox models without stepwise selection. Interaction effects were assessed using subgroup Cox regression. To eliminate immortal time bias, this study employed landmark analysis to evaluate the impact of treatments on overall survival. Landmark time points were set at 3 months and 6 months after diagnosis. The selection was based on both clinical practice cycles and the distribution of treatment initiation times within our study cohort. Specifically, at each landmark point, only patients with an overall survival time ≥ that point were included in the analysis cohort. A patient’s “exposed” status at a landmark was determined based on actual treatment records: if the start time of a specific treatment (e.g., NHA) was on or before the landmark date, the patient was defined as “exposed”; otherwise, they were “unexposed”. Subsequently, the patient’s survival time was reset starting from the landmark point. A multivariable Cox proportional hazards model was then used to analyze the relationship between exposure status and subsequent survival, adjusting for prognostic factors including age, PSA at diagnosis, tumor burden, and baseline skeletal-related events. All analyses were conducted with SPSS 26.0 and R 4.3.1, with p < 0.05 considered statistically significant.

## 3 Results

### 3.1 Incidence of SBM-HSPC and patterns of bone metastasis

**Figure 1:**
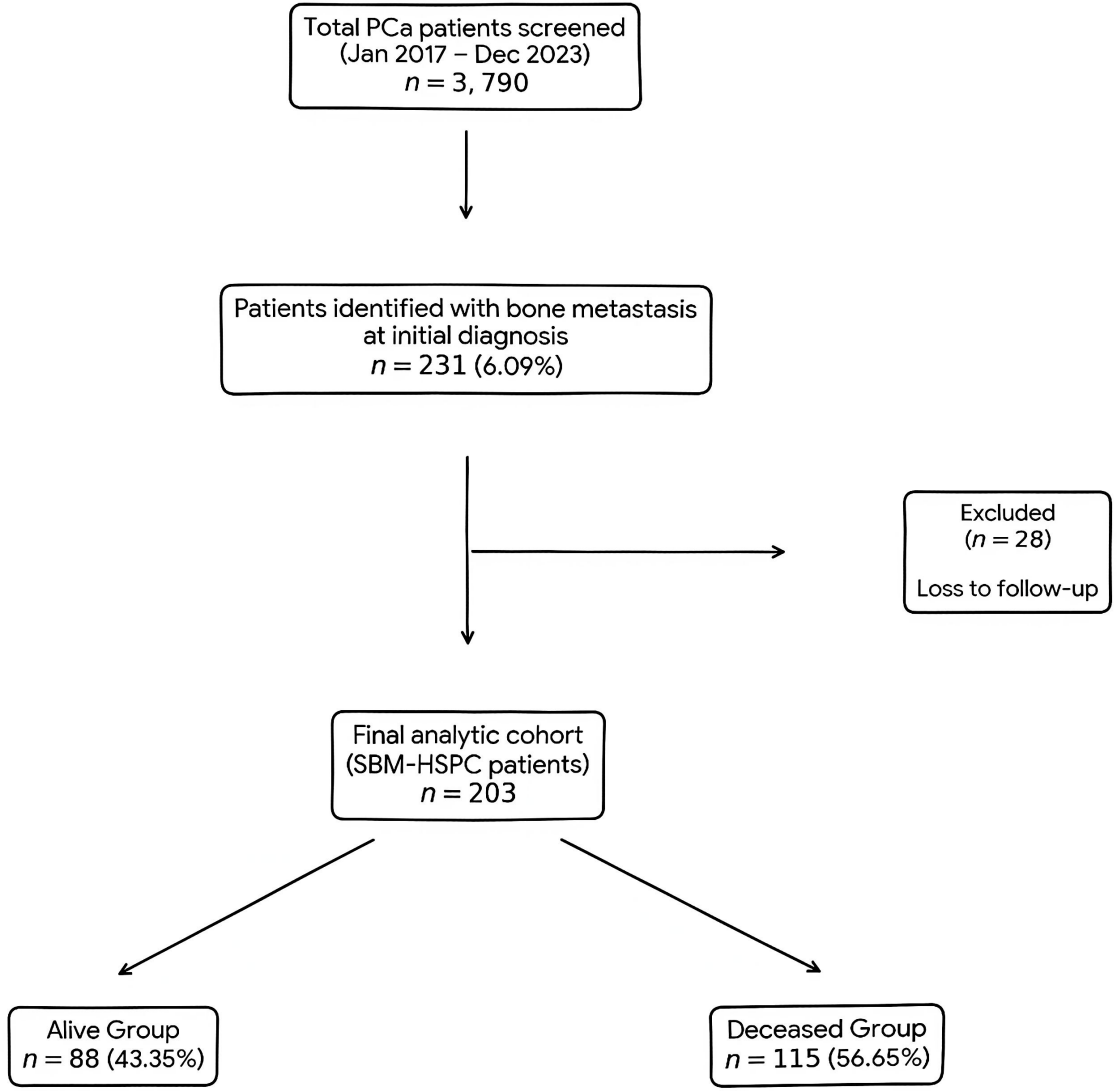
Flowchart of SBM-HSPC patient selection.

A total of 3,790 patients newly diagnosed with prostate cancer (PCa) between 2017 and 2023 at the Affiliated Hospital of Qingdao University were retrospectively reviewed. Among them, 231 patients (6.09%) presented with bone metastasis at initial diagnosis. This figure is comparable to that reported in the North American SEER database (6.8%) but is significantly lower than the rate (30.5%) reported in China in 2014. The median follow-up time for the entire cohort was 36.2 months (interquartile range [IQR]: 20.1 to 58.3 months). After excluding 28 patients due to loss to follow-up, 203 patients were ultimately enrolled in this cohort (**Figure 1**). A comparison of baseline characteristics between patients who completed follow-up (Included group)and those who were lost to follow-up(Excluded group)revealed no significant differences in all key prognostic features, including age, PSA, disease volume, visceral metastasis status, and SREs (**Supplementary Table 1**).

The mean age of the cohort was 70.16 ± 8.15 years, and the mean BMI was 24.01 ± 3.26. Comorbidity analysis revealed that 34.48% (70/203) had hypertension, 16.26% (33/203) had diabetes mellitus, and 7.88% (16/203) had CHD. Regarding behavioral characteristics, 29.56% (60/203) had a history of smoking, and 19.21% (39/203) had a history of regular alcohol consumption. Tumor biological characteristics were highly aggressive: 93.10% (189/203) of patients had a Gleason score ≥ 8, 65.02% (132/203) had a baseline PSA level > 100 ng/mL, and 96.06% (195/203) had PAA as the pathological type. Analysis of metastatic burden indicated that 97.54% (198/203) had axial bone involvement, 83.74% (170/203) met the criteria for HVD, 14.29% (29/203) had concomitant visceral metastasis, and 34.48% (70/203) had experienced SREs. Treatment patterns were characterized by intensive systemic therapy: 89.66% (182/203) received ADT, 51.72% (105/203) received combination therapy with NHA, 33.50% (68/203) received BPs, and 20.69% (42/203) received RANKL. For symptom management, 36.45% (74/203) required opioid analgesic intervention, with 26.60% (54/203) requiring strong opioids or combination regimens.

This cohort, characterized by the triad of high-risk pathology (93.1% with Gleason score ≥ 8), extensive bone metastasis (97.5% with axial involvement), and intensive treatment (over 50% combination therapy with ADT and NHA), is distinctly different from the general prostate cancer population, providing a unique model for exploring specific prognostic factors of bone metastasis.

### 3.2 Characteristics of survival groups in SBM-HSPC

**Figure 2:**
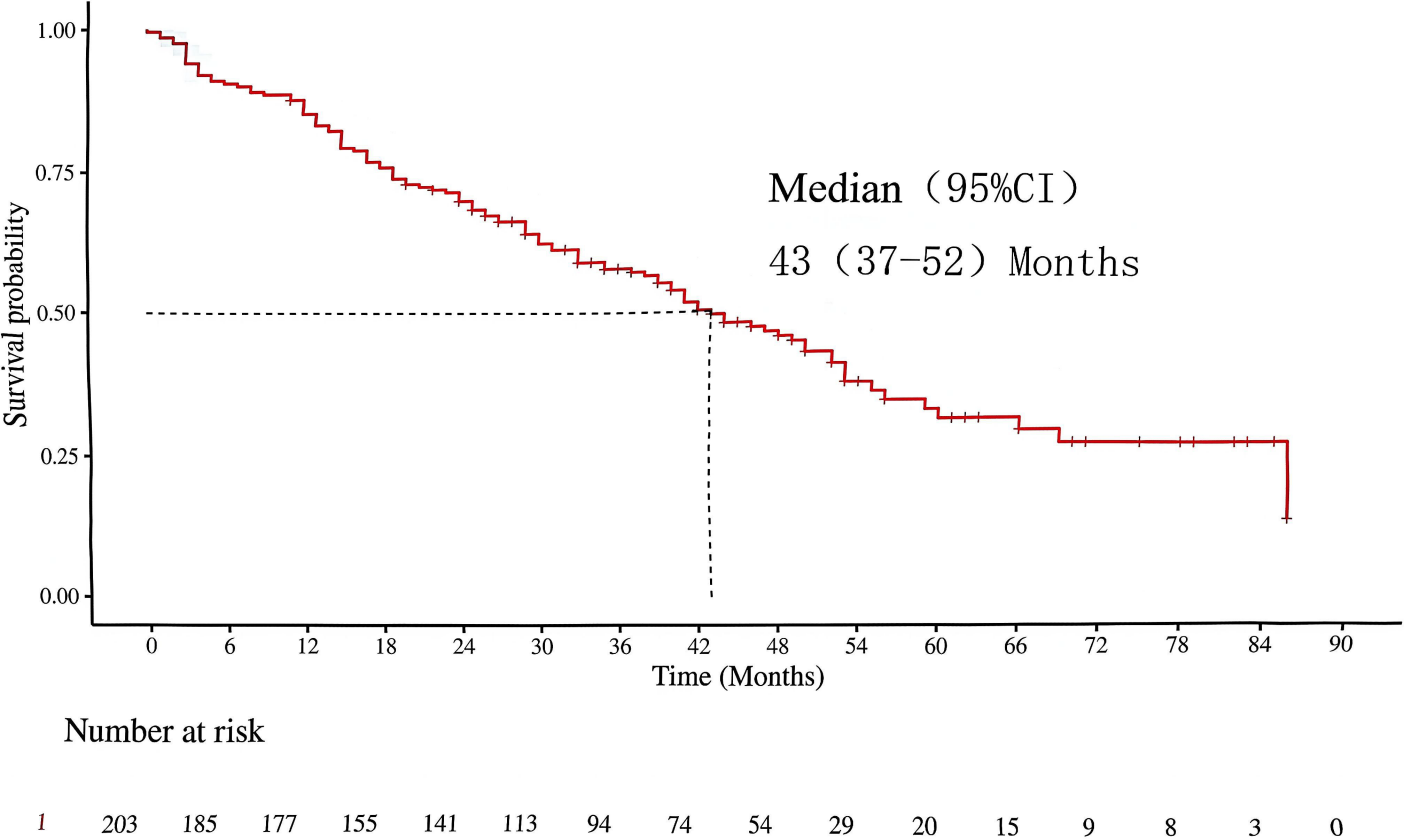
The median survival time of patients with SBM-HSPC.

This study conducted survival follow-up on 203 patients with SBM-HSPC. The median survival time was 43 months (**Figure 2**). Among them, 88 patients (43.35%) were alive (Alive group), and 115 patients (56.65%) had died (Deceased group). Clinical characteristics differed somewhat between the two groups (**Table 1**). Regarding demographic characteristics, the mean age of patients in the deceased group was 71.06 ± 8.93 years, slightly higher than that in the alive group (68.98 ± 6.87 years), but the difference did not reach statistical significance (t = -1.88, P = 0.062). The distribution of smoking history showed a significant difference: the smoking rate in the deceased group was 36.52% (42/115), significantly higher than the 20.45% (18/88) in the alive group (χ² = 6.18, P = 0.013). There were no statistically significant differences in the incidence of comorbidities such as hypertension and diabetes between the two groups. Analysis of metastatic characteristics showed that the incidence of visceral metastasis in the deceased group (18.26%, 21/115) was significantly higher than that in the alive group (7.95%, 7/88) (χ² = 5.09, P = 0.024). The incidence of SREs in the deceased group reached 48.70% (56/115), significantly higher than the 17.05% (15/88) in the alive group (χ² = 20.91, P < 0.001). The proportion of high tumor burden was 84.35% (97/115) in the deceased group and 77.27% (68/88) in the alive group, a difference that was statistically significant (χ² = 4.78, P = 0.029). Comparison of treatment patterns revealed significant distinctions: the utilization rate of BPs in the deceased group (46.96%, 54/115) was significantly higher than that in the alive group (15.91%, 14/88) (χ² = 21.57, P < 0.001). Conversely, the utilization rate of RANKL in the alive group (31.82%, 28/88) was significantly higher than that in the deceased group (12.17%, 14/115) (χ² = 11.72, P < 0.001). NHA were used more frequently in the alive group (62.50%, 55/88) compared to the deceased group (35.65%, 41/115) (χ² = 7.22, P = 0.007), while docetaxel chemotherapy was used more frequently in the deceased group (33.04%, 38/115) compared to the alive group (9.09%, 8/88) (χ² = 7.95, P = 0.005). Pain management data showed that the usage rate of strong opioids (21.74% single use + 13.91% combination) in the deceased group was significantly higher than that in the alive group (6.82% + 2.27%), reflecting a heavier pain symptom burden (χ² = 21.75, P < 0.001). It is noteworthy that the neuroendocrine carcinoma subtype was only present in the deceased group (4.35%, 5/115), while no such pathological type was found in the alive group (P = 0.058). Meanwhile, two seemingly paradoxical phenomena were observed: the BPs usage rate was significantly higher in the deceased group, while the proportion of patients with PSA > 100 ng/mL was higher in the alive group (77.27% vs. 55.65%).

**Table 1:** Baseline Characteristics of all patients.

Variables	Total (n = 203)	Alive (n = 88)	Deceased (n = 115)	P
Age, Mean ± SD	70.16 ± 8.15	68.98 ± 6.87	71.06 ± 8.93	0.062
BMI, Mean ± SD	24.01 ± 3.26	23.99 ± 3.33	24.02 ± 3.22	0.956
HBP, n(%)				0.111
No	133 (65.52)	63 (71.59)	70 (60.87)	
Yes	70 (34.48)	25 (28.41)	45 (39.13)	
Diabetes, n(%)				0.098
No	170 (83.74)	78 (88.64)	92 (80.00)	
Yes	33 (16.26)	10 (11.36)	23 (20.00)	
CHD, n(%)				0.623
No	187 (92.12)	82 (93.18)	105 (91.30)	
Yes	16 (7.88)	6 (6.82)	10 (8.70)	
Smoke, n(%)				0.013
No	143 (70.44)	70 (79.55)	73 (63.48)	
Yes	60 (29.56)	18 (20.45)	42 (36.52)	
Drink, n(%)				0.296
No	164 (80.79)	74 (84.09)	90 (78.26)	
Yes	39 (19.21)	14 (15.91)	25 (21.74)	
PSA(ng/mL), n(%)				0.021
≤4	8 (3.94)	3 (3.41)	5 (4.35)	
(9-10]	7 (3.45)	2 (2.27)	5 (4.35)	
(10-20]	12 (5.91)	2 (2.27)	10 (8.70)	
(20-100]	44 (21.67)	13 (14.77)	31 (26.96)	
>100	132 (65.02)	68 (77.27)	64 (55.65)	
T, n(%)				0.279
T2	90 (44.33)	41 (46.59)	49 (42.61)	
T3	46 (22.66)	23 (26.14)	23 (20.00)	
T4	67 (33.00)	24 (27.27)	43 (37.39)	
N, n(%)				0.413
N0	78 (38.42)	31 (35.23)	47 (40.87)	
N1	125 (61.58)	57 (64.77)	68 (59.13)	
Pathology, n(%)				0.135
PAA	195 (96.06)	87 (98.86)	108 (93.91)	
PDA	3 (1.48)	1 (1.14)	2 (1.74)	
NEPC	5 (2.46)	0 (0.00)	5 (4.35)	
Gleason, n(%)				0.103
≤6	2 (0.99)	0 (0.00)	2 (1.74)	
7	12 (5.91)	8 (9.09)	4 (3.48)	
≥8	189 (93.10)	80 (90.91)	109 (94.78)	
BPs, n(%)				<.001
No	135 (66.50)	74 (84.09)	61 (53.04)	
Yes	68 (33.50)	14 (15.91)	54 (46.96)	
RANKL, n(%)				<.001
No	161 (79.31)	60 (68.18)	101 (87.83)	
Yes	42 (20.69)	28 (31.82)	14 (12.17)	
Analgesics, n(%)				<.001
No analgesic required	129 (63.55)	68 (77.27)	61 (53.04)	
NSAIDs monotherapy	2 (0.99)	0 (0.00)	2 (1.74)	
Weak opioids	23 (11.33)	12 (13.64)	11 (9.57)	
Single-agent strong opioids	31 (15.27)	6 (6.82)	25 (21.74)	
Combination strong opioid therapy	18 (8.87)	2 (2.27)	16 (13.91)	
Axial bone metastasis, n(%)				0.128
No	5 (2.46)	0 (0.00)	5 (4.35)	
Yes	198 (97.54)	88 (100.00)	110 (95.65)	
Visceral metastasis, n(%)				0.024
No	174 (85.71)	81 (92.05)	93 (80.87)	
Yes	29 (14.29)	7 (7.95)	22 (19.13)	
Disease volume, n(%)				0.029
Low	33 (16.26)	20 (22.73)	13 (11.30)	
High	170 (83.74)	68 (77.27)	102 (88.70)	
SREs, n(%)				<.001
No	133 (65.52)	73 (82.95)	60 (52.17)	
Yes	70 (34.48)	15 (17.05)	55 (47.83)	
ADT, n(%)				0.608
No	21 (10.34)	8 (9.09)	13 (11.30)	
Yes	182 (89.66)	80 (90.91)	102 (88.70)	
NHA, n(%)				0.007
No	98 (48.28)	33 (37.50)	65 (56.52)	
Yes	105 (51.72)	55 (62.50)	50 (43.48)	
Docetaxel, n(%)				0.005
No	167 (82.27)	80 (90.91)	87 (75.65)	
Yes	36 (17.73)	8 (9.09)	28 (24.35)	

### 3.3 Survival analysis

**Figure 3:**
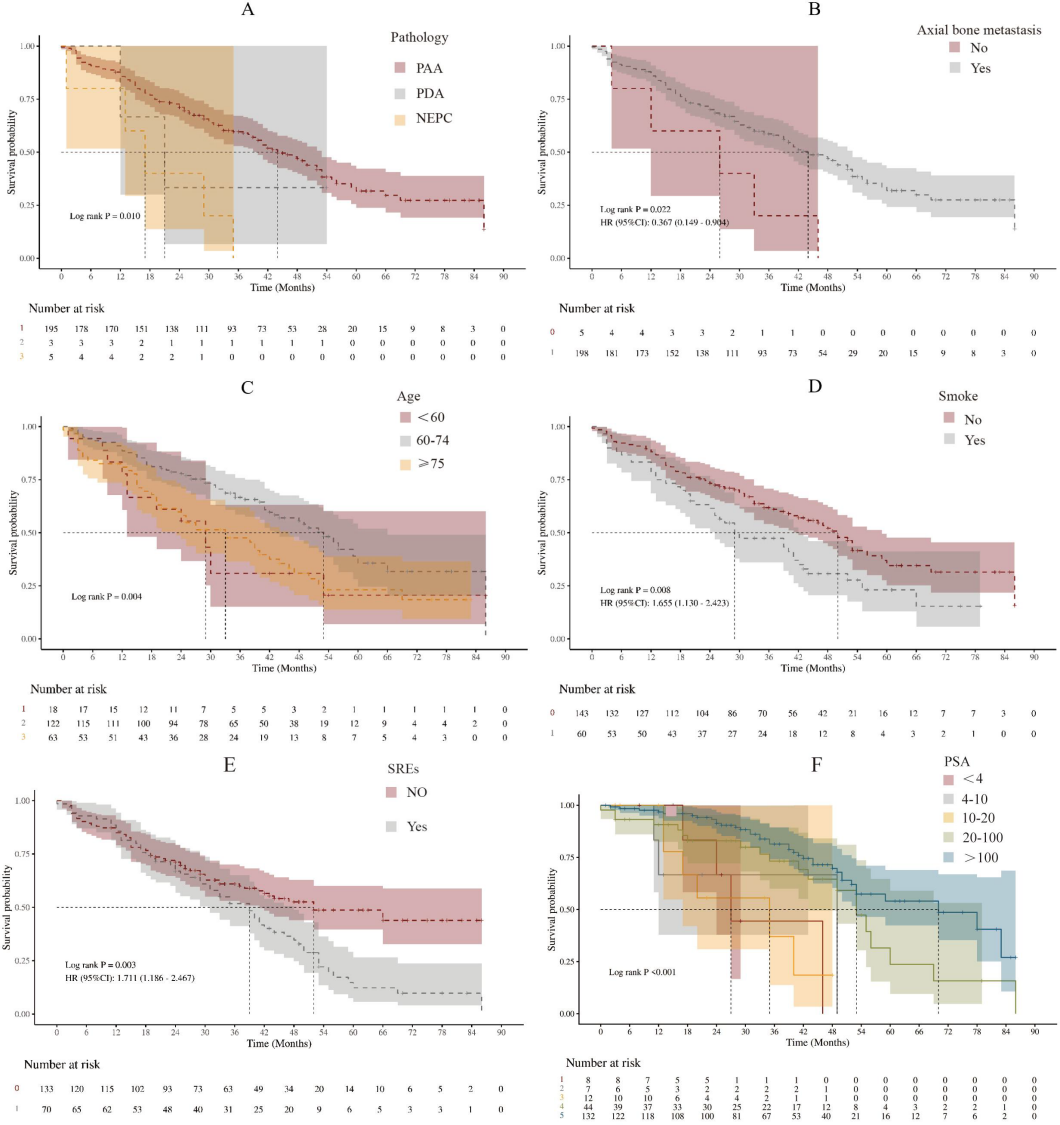
Kaplan-Meier curves of SBM-HSPC patients in different prognostic factors group. **(A)** Pathologytype, **(B)** Axial bone metastasis status, **(C)** Age stratification, **(D)** Smoking statuse, **(E)** SREs, **(F)** PSA.

Kaplan-Meier(KM) survival analysis was initially performed to screen for factors associated with OS in SBM-HSPC patients(**Figure 3**). Variables showing significance (P < 0.05) or deemed clinically essential were selected for further multivariate assessment. The KM analysis preliminarily indicates that pathological type, axial bone metastasis status, age, smoking status, occurrence of SREs, and PSA level at diagnosis are all significantly associated with the overall survival of patients with bone-metastatic prostate cancer. Specifically, smoking and the occurrence of SREs are clear adverse prognostic factors, while the absence of axial bone metastases and age 60–74 years are associated with more favorable survival trends. It is important to note that while PSA level showed significant inter-group survival differences, the specific direction of its association with prognosis requires interpretation alongside subsequent multivariate analysis results. These findings provide a basis for selecting key prognostic variables for inclusion in the subsequent multivariate Cox proportional hazards model.

Univariate and multivariate Cox regression models were employed to identify independent determinants of OS (**Table 2**). In the multivariate model, high disease volume (HDV) emerged as a significant independent risk factor, associated with a 137% increase in the risk of death (HR = 2.37, 95% CI: 1.21–4.64, P = 0.012). Conversely, a diagnostic PSA level ≥ 100 ng/mL was l associated with a lower risk of death compared to the reference group (PSA < 4 ng/mL) (HR = 0.30, 95% CI: 0.11–0.87, P = 0.026). Furthermore, age 60–74 years (vs. <60 years) was associated with a 48% risk reduction (HR = 0.52, 95% CI: 0.28–0.97, P = 0.040), and current smoking increased the risk of death by 52% (HR = 1.52, 95% CI: 1.02–2.27, P = 0.040). The presence of SREs, significant in univariate analysis, lost its independent association in the multivariate model (P = 0.154). The variance inflation factor(VIF) test of the model showed that there was no serious multicollinearity problem in the modified model (**Supplementary Table 2**). To properly account for immortal time bias, the impact of key treatments (NHA, docetaxel, BPs, RANKL inhibitors) was evaluated using landmark analysis at 3 and 6 months post-diagnosis (**Table 3**). After adjusting for baseline prognostic factors, initiation of NHA by the 3-month landmark was associated with a significant 45% reduction in the risk of death (HR = 0.55, 95% CI: 0.39–0.78, P < 0.001). This protective effect remained robust at the 6-month landmark, with a 40% risk reduction (HR = 0.60, 95% CI: 0.42–0.86, P = 0.005). In contrast, early initiation (by 3 or 6 months) of docetaxel chemotherapy, BPs, or RANKL inhibitors did not demonstrate a statistically significant association with overall survival in this cohort.

**Table 2:** Univariate and multivariate Cox regression analyses of OS time.

Variables	Univariate analysis		Multivariate analysis	
	P	HR (95%CI)	P	HR (95%CI)
Age				
<60		1.00 (Reference)		1.00 (Reference)
60-74	**0.034**	0.52 (0.28 ~ 0.95)	**0.040**	0.52 (0.28 ~ 0.97)
≥75	0.836	0.94 (0.50 ~ 1.75)	0.886	1.05 (0.55 ~ 2.00)
Smoke				
No		1.00 (Reference)		1.00 (Reference)
Yes	**0.010**	1.65 (1.13 ~ 2.42)	**0.040**	1.52 (1.02 ~ 2.27)
PSA				
<4		1.00 (Reference)		1.00 (Reference)
4-10	0.743	1.23 (0.36 ~ 4.27)	0.623	1.38 (0.38 ~ 4.97)
10-20	0.495	1.45 (0.50 ~ 4.26)	0.634	1.32 (0.43 ~ 4.06)
20-100	0.447	0.69 (0.27 ~ 1.79)	0.230	0.52 (0.18 ~ 1.51)
≥100	0.091	0.45 (0.18 ~ 1.13)	**0.026**	0.30 (0.11 ~ 0.87)
Disease Volume				
Low		1.00 (Reference)		1.00 (Reference)
High	0.168	1.50 (0.84 ~ 2.68)	**0.012**	2.37 (1.21 ~ 4.64)
SREs				
No		1.00 (Reference)		1.00 (Reference)
Yes	**0.004**	1.71 (1.19 ~ 2.47)	0.154	1.33 (0.90 ~ 1.96)

HR, Hazard Ratio; CI, Confidence Interval. The bold values indicate statistical significance with a p-value < 0.05.

**Table 3:** Cox landmark analysis for treatment.

Landmark point	Treatment	Exposed patients (n, %)	P	HR (95%CI)
3 Months	BPs	15 (7.4%)	0.72	1.12 (0.61 ~ 2.06)
	RANKL	28 (13.7%)	0.52	0.85 (0.52 ~ 1.40)
	NHA	117 (57.4%)	<0.001	0.55 (0.39 ~ 0.78)
	Doce	12 (5.9%)	0.63	1.20 (0.58 ~ 2.48)
6 Months	BPs	28 (14.6%)	0.85	1.05 (0.65 ~ 1.70)
	RANKL	55 (28.6%)	0.60	0.90 (0.61 ~ 1.33)
	NHA	129 (67.2%)	0.005	0.60 (0.42 ~ 0.86)
	Doce	20 (10.4%)	0.86	0.95 (0.52 ~ 1.73)

HR, Hazard Ratio; CI, Confidence Interval.

## 4 Discussion

The biological behavior of malignant tumors dictates their potential for distant metastasis, which is associated with shorter survival. Bones, lungs, and liver are the most common sites of distant metastasis, with approximately 80% of bone metastases originating from breast, lung, and prostate cancers (16). Therefore, investigating the characteristics and prognostic factors of prostate cancer bone metastasis holds significant clinical importance. China has a population of over one billion, yet there has never been a nationwide sampling survey on prostate cancer. The only available data come from a 2008 study focused on developed regions, which may not represent the entire country. Thus, we speculate that the proportion of synchronous bone-metastatic hormone-sensitive prostate cancer (SBM-HSPC) in China in 2008 was significantly higher than the reported 58%. Among the more than 300 cities in mainland China, Beijing, Shanghai, and Guangzhou (the three cities included in the 2008 urology center study) consistently rank among the top four in terms of GDP. Our center is located in Qingdao, which ranked between 12th and 14th nationally from 2017 to 2023, making it representative of developed regions in China. This study is the first to report a prevalence of 6.09% for SBM-HSPC at a tertiary referral center in China, which is significantly lower than the previously reported 54% in multi-center studies and closely aligns with the 6.8% reported in the North American SEER database (13). Our study noted a marked temporal shift in the proportion of patients diagnosed with SBM-HSPC, decreasing from approximately 54% to 6% over the study period. While this trend is intriguing, its drivers in our single-center, retrospective cohort remain speculative and are likely multifactorial. Several concurrent developments could plausibly contribute to this observation. First, it coincided with broader advancements in prostate cancer management, including increased public awareness, potential shifts in PSA screening practices (17–19), and the adoption of more sensitive staging imaging (e.g., PSMA-PET/CT), which may have led to earlier diagnosis and stage migration. Second, changes in local referral patterns or patient demographics at our institution over time could have influenced the case mix. Finally, we cannot rule out the possibility of evolving diagnostic thresholds or documentation practices in electronic health records. Crucially, our study design did not capture direct data on screening rates, referral criteria, or imaging utilization patterns; therefore, we cannot ascertain the relative contribution of these factors or establish causality. The observed decline may represent a combination of improved early detection and genuine shifts in disease presentation. Despite these limitations, the dramatic reduction in the incidence of SBM-HSPC at diagnosis, if validated in multi-center or population-level studies, could have significant implications for prognostic stratification and initial treatment planning. Future prospective studies incorporating detailed data on diagnostic pathways are needed to elucidate the precise reasons behind this temporal trend.

Previous studies have suggested that the timing of metastasis is a critical concept in mCSPC patients. Those with synchronous mCSPC had a 5-year OS rate of 39%, significantly lower than the 79% observed in patients with metachronous mCSPC (P < 0.01). Additionally, synchronous mCSPC patients exhibited lower androgen receptor pathway activity (5, 8). Therefore, we speculate that SBM-HSPC may demonstrate higher aggressiveness and poorer prognosis compared to the more common metachronous mHSPC population. In this cohort, the median OS of SBM-HSPC patients was 42 months. Among subgroups, those untreated with NHA had a median OS of 33 months, whereas those treated with NHA reached a median OS of 52 months. This outcome is only comparable to the therapeutic effect observed in the LATITUDE study (median OS of 36.5 months in the ADT-alone group vs. 53.3 months in the ADT plus abiraterone group among high-risk mHSPC patients) (11). In this cohort, 93.1% of patients had a Gleason score ≥ 8, which is close to the proportion in the high-risk group of the TITAN study (96.5–97.9%) and considerably higher than that in the low-risk group (30.5–32.4%), confirming that SBM-HSPC patients predominantly exhibit highly aggressive tumor biological behavior (11).

This study identified HDV (HR = 2.38, P = 0.013) as the most robust independent risk factor in the SBM-HSPC population. While the CHAARTED study reported high tumor burden in 65% of its cohort (15), in our cohort, 83.74% of patients were classified as HDV, which intuitively reflects the highly aggressive and metastatic characteristics of this SBM-HSPC patient group. Consequently, the median OS for HDV patients was 42 months, whereas the median OS for LDV patients has not yet been reached. Given these findings, early intensive therapy (such as an ADT + novel endocrine agent + chemotherapy triple regimen) appears more suitable as a standard choice for the HDV subgroup within SBM-HSPC patients. Among other prognostic factors, smoking (HR = 1.48, P = 0.061) demonstrated borderline significance, representing a strong trend. It is well-established that smoking can facilitate cancer progression by promoting inflammation, suppressing immunity, and inducing genetic mutations, thereby creating a more hostile “microenvironment” for cancer. This finding is corroborated by previous research: current smokers have an increased risk of fatal prostate cancer (RR = 1.14; 95% CI = 1.06–1.19), with the heaviest smokers facing a 24% to 30% higher risk of dying from prostate cancer compared to non-smokers (20). Furthermore, smoking prostate cancer patients have an 89% higher risk of death, a 151% higher risk of metastasis, and a 40% higher risk of postoperative PSA recurrence compared to their non-smoking counterparts (21).

A notable finding from our multivariate analysis was that a diagnostic PSA level ≥100 ng/mL was associated with improved OS. We interpret this counterintuitive observation with considerable caution, as it is likely influenced by several important limitations inherent to our study design and data. Crucially, our analysis was constrained by the nature of the available PSA data. For the majority of patients (>70%) with PSA >100 ng/mL, only categorical data were recorded, lacking precise numerical values. This fundamental data limitation precluded modeling PSA as a continuous variable (e.g., using log-PSA with splines), which is the optimal method for exploring its potentially complex, non-linear relationship with survival. Consequently, the observed association may be sensitive to the specific categorization thresholds used. Furthermore, this association could be confounded by unmeasured or residual factors. It may partly reflect the inclusion of aggressive tumor variants that present with disproportionally low PSA levels. Differences in diagnostic intensity or staging workup across patients could also introduce bias. Most importantly, our model lacked several key prognostic covariates, such as performance status and a more granular assessment of metastatic burden, whose absence may distort the estimated effect of PSA. Therefore, we emphasize that our results describe a statistical association, not a causal or “protective” effect. The very high PSA level is more accurately viewed as a composite marker that may correlate with a specific tumor biology (e.g., androgen receptor pathway dominance), prompt clinical decision-making leading to earlier intensive therapy, and/or residual confounding (22). This finding should be considered hypothesis-generating and underscores the complexity of prognostic stratification in metastatic prostate cancer. Future studies with prospectively collected, continuous PSA data and comprehensive biomarker panels are needed to clarify the true prognostic role of extreme PSA elevations.

The landmark analysis conducted at 3 and 6 months post-diagnosis provides a methodologically robust assessment of treatment effects while mitigating immortal time bias. The central and most consistent finding is the significant association between early initiation of Novel Hormonal Agents (NHA) and improved overall survival. Patients who had commenced NHA therapy within 3 months of diagnosis experienced a 45% reduction in the risk of death (HR = 0.55, P<0.001), and this protective association remained strong at the 6-month landmark, with a 40% risk reduction (HR = 0.60, P = 0.005). This finding reinforces the pivotal role of NHA in the first-line setting for metastatic hormone-sensitive prostate cancer (mHSPC), aligning with the survival benefits demonstrated in pivotal trials such as TITAN and ENZAMET (11, 23). Our data suggest that the survival advantage is maximized when treatment is initiated promptly, underscoring the importance of timely therapeutic intervention.

In contrast, early initiation (within 3–6 months) of other systemic therapies—namely docetaxel chemotherapy (Doce), bisphosphonates (BPs), and RANKL inhibitors—was not associated with a statistically significant improvement in overall survival in this cohort. Several non-mutually exclusive explanations may account for these observations. For docetaxel, the lack of a significant association may be influenced by the selected patient population, treatment sequencing relative to NHA, or the sample size of the exposed group, which was relatively small (n=12 at 3 months, n=20 at 6 months), potentially limiting statistical power to detect an effect. Regarding bone-targeting agents (BPs and RANKL inhibitors), their primary clinical objective is the prevention of skeletal-related events (SREs) rather than the direct extension of overall survival. While they are cornerstone therapies for bone metastases, a pronounced survival benefit in an all-comer population may not be evident, especially in a study not primarily designed to assess SREs as an endpoint. Our results highlight the distinct mechanisms and goals of these therapeutic classes.

This current study revealed that PC patients aged 60–74 had a significantly lower risk of death—46% reduction (HR = 0.54, P = 0.056)—compared to those under 60. This finding is highly consistent with the “J-shaped age-risk curve” reported by Humphreys et al., whose research showed that the median survival in the 65–74 age group reached 7.8 years, significantly longer than that of the <55 group (5.5 years) and the ≥75 group (4.3 years) (24). This survival advantage may stem from a dual mechanism: on the one hand, younger patients often present with more aggressive tumors (such as a higher proportion of initial diagnoses at stage IV and earlier bone metastases) and may carry susceptibility genes like BRCA; on the other hand, patients aged 60–74 combine favorable treatment tolerance with a relatively long hormone-sensitive period (median of 21.5 months), enabling them to benefit more effectively from intensive therapies. Although the result did not reach the traditional threshold of statistical significance, the strong trend effect (upper CI of 1.02) suggests that ages 60–74 may represent a “golden window” for survival, which holds important clinical implications for innovating age-based stratified treatment strategies.

Through this study, we have recognized that SBM-HSPC patients are not a homogeneous group, and patient stratification is the cornerstone of scientific management. The ARASENS study in 2022 confirmed that the ADT + NHA + chemotherapy triple therapy significantly reduced the risk of death in mHSPC patients, promoting the triple therapy to become a first-line treatment option (25), particularly recommended for patients with high tumor burden, high-risk, and newly diagnosed cases who can tolerate docetaxel (26). Additionally, this study reveals the complexity of treatment choices in the real world. Clinicians should be aware that for patients in suitable physical condition, proven survival-improving drugs such as NHA and RANKL inhibitors should be used more aggressively, rather than being reserved only for patients with “better expectations,” thereby avoiding a “self-fulfilling prophecy.” Certainly, our research is subject to several limitations. Firstly, as a retrospective analysis, it cannot entirely rule out treatment selection bias. Secondly, the lack of molecular mechanism validation limits the depth of our conclusions. Thirdly, the unstable estimates for certain subgroups—such as the intermediate Gleason score—likely reflect limited sample sizes in these categories, warranting further validation in larger cohorts. Finally, the use of single-center data significantly restricts the generalizability of our findings.

## 5 Conclusion

This retrospective cohort study delineates the clinical profile, prognostic determinants, and treatment outcomes of patients with SBM-HSPC at a major Chinese tertiary center. The observed incidence of 6.09% aligns more closely with contemporary data from developed healthcare systems than with prior national estimates, suggesting a potential shift in disease presentation within China’s developed regions, though the specific drivers require further investigation. The prognosis of SBM-HSPC is dictated by a confluence of tumor biology, disease burden, and therapeutic intervention. We identified HDV as the most robust independent risk factor for mortality, underscoring the aggressive nature of this presentation. The early initiation of NHA—within 3 to 6 months of diagnosis—was strongly and consistently associated with a significant survival benefit, reinforcing the critical importance of timely, intensive systemic therapy in this setting. In contrast, early use of other therapies, including docetaxel and bone-targeting agents, was not associated with improved overall survival in this cohort, highlighting the distinct roles of these treatments. The study also revealed nuanced associations: smoking was linked to increased mortality risk, while patients aged 60–74 years exhibited a more favorable survival trend compared to younger patients, possibly indicating a “therapeutic window” of optimal treatment tolerance and benefit. The counterintuitive association between an extremely high diagnostic PSA (≥100 ng/mL) and better survival warrants extreme caution in interpretation. It likely represents a complex signal influenced by unmeasured confounding, specific tumor biology, and clinical management pathways, rather than a direct protective effect. In summary, SBM-HSPC represents a distinct, high-risk subset of prostate cancer. Management should prioritize immediate risk stratification, focusing on disease volume, and the expeditious commencement of NHA-based combination therapy. The findings advocate for a stratified treatment approach and highlight the need for prospective, multi-center studies to validate these associations, refine prognostic models, and optimize therapeutic sequences for this challenging patient population.

## Data Availability

The raw data supporting the conclusions of this article will be made available by the authors, without undue reservation.

## Supplementary material

Supplementary Table 1 Baseline characteristics of included and excluded patients. SD, standard deviation.

Supplementary Table 2 The variance inflation factor (VIF) test of the model

## References

[1] Raychaudhuri R Lin DW Montgomery RB . Prostate cancer: a review. Jama. (2025) 333:1433–46. doi: 10.1001/jama.2025.7193, PMID: 40063046

[2] Rebello RJ Oing C Knudsen KE Loeb S Johnson DC Reiter RE . Prostate cancer. Nat Rev Dis Primers. (2021) 7:9. doi: 10.3390/genes8020071. PMID: 33542230

[3] Sartor O . Localized prostate cancer - then and now. N Engl J Med. (2023) 388:1617–8. doi: 10.1056/nejme2300807. PMID: 36912567

[4] Cheng B He H Chen B Zhou Q Luo T Li K . Assessment of treatment outcomes: cytoreductive surgery compared to radiotherapy in oligometastatic prostate cancer - an in-depth quantitative evaluation and retrospective cohort analysis. Int J Surg (London England). (2024) 110:3190–202. doi: 10.1097/js9.0000000000001308. PMID: 38498388 PMC11175786

[5] Antonarakis ES Shui IM Zaidi O Bernauer M Gratzke C . Current treatment paradigms and clinical outcomes in oligometastatic prostate cancer patients: a targeted literature review. Eur Urol Oncol. (2024) 7:1280–92. doi: 10.1016/j.euo.2024.06.002. PMID: 38964996

[6] Finianos A Gupta K Clark B Simmens SJ Aragon-Ching JB . Characterization of differences between prostate cancer patients presenting with De Novo versus primary progressive metastatic disease. Clin Genitourin Cancer. (2017) 16:85–90. doi: 10.1016/j.clgc.2017.08.006. PMID: 28899723

[7] Markowski MC Ren Y Tierney M Royce TJ Yamashita R Croucher D . Digital pathology-based artificial intelligence biomarker validation in metastatic prostate cancer. Eur Urol Oncol. (2025) 8:755–62. doi: 10.1200/jco.2024.42.16_suppl.5077. PMID: 39665917 PMC12369405

[8] Sutera PA Shetty AC Hakansson A Van der Eecken K Song Y Liu Y . Transcriptomic and clinical heterogeneity of metastatic disease timing within metastatic castration-sensitive prostate cancer. Ann Oncol Off J Eur Soc For Med Oncol. (2023) 34:605–14. doi: 10.1016/j.ijrobp.2023.06.1217. PMID: 37164128 PMC10330666

[9] Roubaud G Kostine M McDermott RS Bernard-Tessier A Maldonado X Silva M . Assessment of bone mineral density in men with de novo metastatic castration-sensitive prostate cancer treated with or without abiraterone acetate plus prednisone in the PEACE-1 phase 3 trial. Eur J Cancer (Oxford England: 1990). (2025) 218:115293. doi: 10.1016/j.ejca.2025.115293. PMID: 39923274

[10] Sydes MR Spears MR Mason MD Clarke NW Dearnaley DP de Bono JS . Adding abiraterone or docetaxel to long-term hormone therapy for prostate cancer: directly randomised data from the STAMPEDE multi-arm, multi-stage platform protocol. Ann Oncol Off J Eur Soc For Med Oncol. (2018) 29:1235–48. doi: 10.1093/annonc/mdy072. PMID: 29529169 PMC5961425

[11] Fizazi K Tran N Fein L Matsubara N Rodriguez-Antolin A Alekseev BY . Abiraterone acetate plus prednisone in patients with newly diagnosed high-risk metastatic castration-sensitive prostate cancer (LATITUDE): final overall survival analysis of a randomised, double-blind, phase 3 trial. Lancet Oncol. (2019) 20:686–700. doi: 10.1016/s1470-2045(19)30082-8. PMID: 30987939

[12] Fizazi K Foulon S Carles J Roubaud G McDermott R Fléchon A . Abiraterone plus prednisone added to androgen deprivation therapy and docetaxel in de novo metastatic castration-sensitive prostate cancer (PEACE-1): a multicentre, open-label, randomised, phase 3 study with a 2 × 2 factorial design. Lancet (London England). (2022) 399:1695–707. doi: 10.1016/s0140-6736(22)00367-1. PMID: 35405085

[13] Ma C Ye D Li C Zhou F Yao X Zhang S . Epidemiological characteristics and first-line endocrine therapy for advanced prostate cancer(in Chinese). Chin J Surg. (2008) 46:921–5. doi: 10.3321/j.issn:0529-5815.2008.12.012, PMID: 19035151

[14] Desai MM Cacciamani GE Gill K Zhang J Liu L Abreu A . Trends in incidence of metastatic prostate cancer in the US. JAMA Netw Open. (2022) 5:e222246. doi: 10.1001/jamanetworkopen.2022.2246. PMID: 35285916 PMC9907338

[15] Sweeney CJ Chen YH Carducci M Liu G Jarrard DF Eisenberger M . Chemohormonal therapy in metastatic hormone-sensitive prostate cancer. N Engl J Med. (2015) 373:737–46. doi: 10.1056/nejmoa1503747. PMID: 26244877 PMC4562797

[16] Coleman RE Croucher PI Padhani AR Clézardin P Chow E Fallon M . Bone metastases. Nat Rev Dis Primers. (2020) 6:83. doi: 10.1016/j.breast.2011.08.023. PMID: 33060614

[17] Kitagawa Y Namiki M . Prostate-specific antigen-based population screening for prostate cancer: current status in Japan and future perspective in Asia. Asian J Andrology. (2015) 17:475–80. doi: 10.4103/1008-682x.143756. PMID: 25578935 PMC4430954

[18] Ramsey SD Bansal A Fedorenko CR Blough DK Overstreet KA Shankaran V . Financial insolvency as a risk factor for early mortality among patients with cancer. J Clin Oncol Off J Am Soc Clin Oncol. (2016) 34:980–6. doi: 10.1200/jco.2015.64.6620. PMID: 26811521 PMC4933128

[19] Hugosson J Roobol MJ Månsson M Tammela TLJ Zappa M Nelen V . A 16-yr follow-up of the European randomized study of screening for prostate cancer. Eur Urol. (2019) 76:43–51. doi: 10.1590/s1677-5538.ibju.2020.03.06. PMID: 30824296 PMC7513694

[20] Huncharek M Haddock KS Reid R Kupelnick B . Smoking as a risk factor for prostate cancer: a meta-analysis of 24 prospective cohort studies. Am J Public Health. (2010) 100:693–701. doi: 10.2105/ajph.2008.150508. PMID: 19608952 PMC2836346

[21] Foerster B Pozo C Abufaraj M Mari A Kimura S D'Andrea D . Association of smoking status with recurrence, metastasis, and mortality among patients with localized prostate cancer undergoing prostatectomy or radiotherapy: a systematic review and meta-analysis. JAMA Oncol. (2018) 4:953–61. doi: 10.1001/jamaoncol.2018.1071. PMID: 29800115 PMC6145736

[22] Halin Bergström S Semenas J Nordstrand A Thysell E Wänman J Crnalic S . Morphological heterogeneities in prostate cancer bone metastases are related to molecular subtypes and prognosis. Clin Exp Metastasis. (2025) 42:49. doi: 10.1007/s10585-025-10365-y. PMID: 40841830 PMC12370827

[23] Sweeney CJ Martin AJ Stockler MR Begbie S Cheung L Chi KN . Testosterone suppression plus enzalutamide versus testosterone suppression plus standard antiandrogen therapy for metastatic hormone-sensitive prostate cancer (ENZAMET): an international, open-label, randomised, phase 3 trial. Lancet Oncol. (2023) 24:323–34. doi: 10.1016/s1470-2045(23)00063-3. PMID: 36990608

[24] Humphreys MR Fernandes KA Sridhar SS . Impact of age at diagnosis on outcomes in men with castrate-resistant prostate cancer (CRPC). J Cancer. (2013) 4:304–14. doi: 10.7150/jca.4192. PMID: 23569463 PMC3619091

[25] Smith MR Hussain M Saad F Fizazi K Sternberg CN Crawford ED . Darolutamide and survival in metastatic, hormone-sensitive prostate cancer. N Engl J Med. (2022) 386:1132–42. doi: 10.1056/nejmoa2119115. PMID: 35179323 PMC9844551

[26] Hussain M Fizazi K Shore ND Heidegger I Smith MR Tombal B . Metastatic hormone-sensitive prostate cancer and combination treatment outcomes: a review. JAMA Oncol. (2024) 10:807–20. doi: 10.1001/jamaoncol.2024.0591. PMID: 38722620

