# Supplementary material for: Early Initiation of novel hormonal therapy is associated with improved survival in synchronous bone-metastatic hormone-sensitive prostate cancer: a retrospective cohort study from China
Source: Front Oncol. 2026 Apr 1;16:1719338. doi: 10.3389/fonc.2026.1719338 (PMC13079022; doi:10.3389/fonc.2026.1719338)
Supplement: Supplementary Table 1 — Baseline characteristics of included and excluded patients. SD, standard deviation. [file Table1.docx]

Table S1 Baseline Characteristics of included and excluded patients

| Variables | Total (n = 231) | Included (n = 203) | Excluded (n = 28) | P |
| --- | --- | --- | --- | --- |
| Age, Mean ± SD | 70.26 ± 8.18 | 70.16 ± 8.15 | 71.04 ± 8.52 | 0.596 |
| PSA(ng/mL), n(%) |  |  |  | 0.081 |
| ≤4 | 12 (5.19) | 8 (3.94) | 4 (14.29) |  |
| (9-10] | 8 (3.46) | 7 (3.45) | 1 (3.57) |  |
| (10-20] | 15 (6.49) | 12 (5.91) | 3 (10.71) |  |
| (20-100] | 51 (22.08) | 44 (21.67) | 7 (25.00) |  |
| ＞100 | 145 (62.77) | 132 (65.02) | 13 (46.43) |  |
| Visceral metastasis, n(%) |  |  |  | 0.825 |
| No | 199 (86.15) | 174 (85.71) | 25 (89.29) |  |
| Yes | 32 (13.85) | 29 (14.29) | 3 (10.71) |  |
| Disease volume, n(%) |  |  |  | 1.000 |
| Low | 38 (16.45) | 33 (16.26) | 5 (17.86) |  |
| High | 193 (83.55) | 170 (83.74) | 23 (82.14) |  |
| SREs, n(%) |  |  |  | 0.535 |
| No | 153 (66.23) | 133 (65.52) | 20 (71.43) |  |
| Yes | 78 (33.77) | 70 (34.48) | 8 (28.57) |  |

SD: standard deviation
