# Supplementary material for: Early Initiation of novel hormonal therapy is associated with improved survival in synchronous bone-metastatic hormone-sensitive prostate cancer: a retrospective cohort study from China
Source: Front Oncol. 2026 Apr 1;16:1719338. doi: 10.3389/fonc.2026.1719338 (PMC13079022; doi:10.3389/fonc.2026.1719338)
Supplement: Supplementary Table 2 — The variance inflation factor (VIF) test of the model [file Table2.docx]

Table S2 The variance inflation factor（VIF） test of the model

Variables GVIF

| Age | 1.073 |
| --- | --- |
| Smoke | 1.060 |
| PSA | 1.284 |
| DV | 1.191 |
| SREs | 1.054 |
